# Immigrant Perspectives of Social Connection in a Nontraditional Migration Area

**DOI:** 10.3390/healthcare12060686

**Published:** 2024-03-19

**Authors:** Farrah Jacquez, Lisa M. Vaughn, Jamie Hardy-Besaw

**Affiliations:** 1 Department of Psychology, University of Cincinnati, Cincinnati, OH 45221, USA; besawjd@mail.uc.edu; 2 Cincinnati Children’s Hospital Medical Center, College of Medicine, University of Cincinnati, Cincinnati, OH 45221, USA; lisa.vaughn@cchmc.org

**Keywords:** connectedness, social ecological, ecological systems, belonging, social support, social capital, navigation, social acceptance, immigrant health

## Abstract

Social connection is a core dimension of health and wellness among all populations, yet the experience of moving to and living in a new country makes social and community-level influences particularly salient for immigrants. We interviewed 38 Latino immigrants living in a nontraditional migration area to explore the social and community foundations of health and wellness. Using hybrid (inductive/deductive) qualitative analysis, we identified seven domains of social connection from the perspective of the interviewed participants: (1) lens of the individual; (2) immigrant experience; (3) interpersonal support; (4) community belonging; (5) community capital; (6) community navigation; and (7) social acceptance. Social connection domains generated by participants are consistent with the scientific literature, but this study identifies the specific social factors that immigrants describe as most salient to their own health and wellness. Our community-generated understanding of social connection can be used by healthcare providers to reduce risks and build on assets that will improve the health of immigrants living in nontraditional migration areas. Additionally, these results might serve as a foundation for a quantitative measure that can be used by providers to more accurately and comprehensively assess the social connection of their patients and by researchers to evaluate the effectiveness of community-level interventions for immigrants.

## 1. Introduction

More than 44.9 million people in the United States (~14% of the population) are immigrants [1]. Compared to other countries, the US hosts the largest number of foreign-born people [1,2], and these populations continue to increase, particularly in nontraditional migration areas such as the Midwest [3,4]. US immigrants, compared to native-born populations, experience significant mental and physical health disparities [5,6,7,8,9,10] and a multitude of stressors due to unique psychosocial, environmental, socioeconomic, migration, and acculturative challenges [11,12,13,14,15], all of which contribute to negative psychological and biological health impacts over the life-course including an increased risk of chronic disease, suicide, addiction, and early death [8,16,17,18,19]. Inadequacies in access and quality of healthcare further impact immigrant health outcomes. Immigrants are less likely to utilize healthcare [20], to have difficulty accessing healthcare systems [21], and to perceive inequitable treatment by providers [22,23,24,25].

Efforts to address immigrant health disparities have traditionally focused on individual differences in language, acculturation, legal status, and health behaviors [26], despite widespread recognition of the salient social determinants driving health outcomes. The socio-ecological model pioneered by Bronfenbrenner [27,28] and expanded by researchers over the last two decades [29,30,31,32] emphasizes the complex interplay between individual, relationship, community, and societal systems and their role in determining health outcomes. Research that has gone beyond the individual level in immigrant health includes some consideration of interpersonal and community-level factors, but the literature is diffuse and difficult to distill into distinct mechanisms [33,34]. Societal-level variables have gained more attention in the past decade, including the impact of both state and federal immigration policies on healthcare utilization [35] and health outcomes [36]. The crucial next step toward immigrant health equity is accurately understanding social connections across ecological systems.

Social disconnection is associated with decrements and disparities in physical and mental health for vulnerable and minoritized groups such as immigrants [37,38,39,40]. Notably, the Hispanic health paradox shows more positive health advantages for Latino immigrants, but these associations are actually moderated by social networks [41]. In other words, the Hispanic health paradox is dependent on the number and quality of social connections. Social processes influence health outcomes among all populations, but the experience of moving to, and living in, a new country makes social and community-level influences particularly salient for immigrants. Previous literature has identified social connections in the interpersonal, familial, and community realms as primary mechanisms driving health in immigrants [42,43,44,45,46], but the specific processes are not well-defined. Social support has a robust base of evidence for positive associations with immigrant physical and mental health [14,47,48,49,50], but likewise, the concept of social support is diffused, with operationalizations ranging from family support [51] to social cohesion [52] to engagement in online forums [53]. Social networks have been identified as a protective factor against depression, anxiety, and substance use disorders in immigrants [54] and have been theorized to be the link between cultural processes and positive health outcomes [41]. Social capital has also been associated with better physical and mental health and improved quality of life in immigrants [55,56,57,58]. Community-level variables like belonging [59,60,61] and neighborhood social cohesion [62,63] also play a role in positive health outcomes for immigrants.

One protective factor experienced by Latino immigrants is the support provided by well-established social networks in the ethnic enclaves of large urban areas and border states [64,65]. In comparison, immigrants living in nontraditional migration areas, or parts of the country that have not typically been home to a significant population of migrants [66,67,68,69,70] (also known in the literature as nontraditional receiving sites [71], emerging destinations [49,72,73,74,75], new migration areas [76,77], and newcomer populations/areas [78,79,80]) are more likely to lack familial and cultural social ties [81]. In our local context of Greater Cincinnati, the Latino population grew 91% between 2000 and 2020 [82,83]. The sudden exponential growth of Latinx immigrants in Cincinnati creates a social ecology that mirrors other emerging migration areas. The human service institutions in our area have struggled to adapt to the unique needs of Latinx families, particularly in the context of policies that exclude many immigrants from receiving services [84]. Whereas large urban areas and border states tend to have ethnic enclaves that facilitate social support [6], Greater Cincinnati does not have the historical immigration precedent to form well-established social networks [85]. In healthcare, growth in Spanish-speaking providers has not matched the demand, so there is a major shortage of linguistically and culturally appropriate care. In the metro area outside the city center, anti-immigrant sentiment, highly publicized immigration raids, and state-wide policies restricting employment and driving privileges for undocumented immigrants make many Latino families reluctant to engage in their communities. In our previous work, we found low perceived social acceptance among Cincinnati Latinos, and those who reported lower social acceptance had worse mental health [68]. To accurately understand how social connection influences immigrant health, research must include the nontraditional migration areas in the South and Midwest, which are the largest growth areas for Latino migration [86,87,88,89].

Based on compelling evidence linking social isolation and early death [90], the U.S. Surgeon General described social disconnection as a public health epidemic. His *2023 Advisory Report on the Healing Effects of Social Connection and the Community* called on researchers to elucidate intersections between social connection indicators that influence risk and resilience for health outcomes [91]. The everyday social environment of immigrants, particularly those living outside the biggest cities and border states, can make them particularly disconnected and, therefore, vulnerable. Emerging migration areas tend to lack ethnic enclaves and linguistically appropriate social support services that can serve as protective factors, so the risk for social disconnection in these areas is especially high. Identifying the specific aspects of the social environment that are most salient for immigrants is critical in developing interventions and improving healthcare systems. In this study, we aimed to conceptualize the social ecology of immigrants or the aspects of the psychosocial environment that might serve as social determinants of health [92]. Because immigrants living in nontraditional migration areas may have social connection experiences that are not captured by the existing literature, we take a qualitative approach that will allow immigrants to define social connection from their own perspective. Using a hybrid inductive/deductive analytic approach, our research objective was to operationalize the concept of social connection of immigrants from the experiences and perspectives of immigrants themselves.

## 2. Materials and Methods

We conducted a qualitative interview study using a hybrid (inductive/deductive) process of thematic analysis [93,94,95,96,97,98,99]. Because the primary purpose of the study was to inform local interventions and advocacy, our university’s institutional review board determined the study was not human subjects research. Data collection and analysis procedures were co-designed with community partners to protect participants and ensure optimal relevance to community needs.

### 2.1. Participants and Community Partners

We interviewed 38 Latino immigrants living in the Greater Cincinnati region (including neighboring regions of Indiana and Kentucky). Note that we use “Latino” throughout this manuscript as it is the preferred term of our community partners. All participants were immigrants from Latin America, 18 years or older, and spoke English, Spanish, or Ixil (a native Mayan language spoken in Mexico and Guatemala). See Table 1 for participant demographic information. The research team included four members of Latinos Unidos por la Salud (LU-Salud), an established Latino community research team working to improve health equity in Cincinnati [66,77,100,101,102]. LU-Salud partners received training in qualitative research and were involved in the design, preparation, data collection, interpretation, and dissemination phases of the study.

### 2.2. Setting

**Figure 1:**
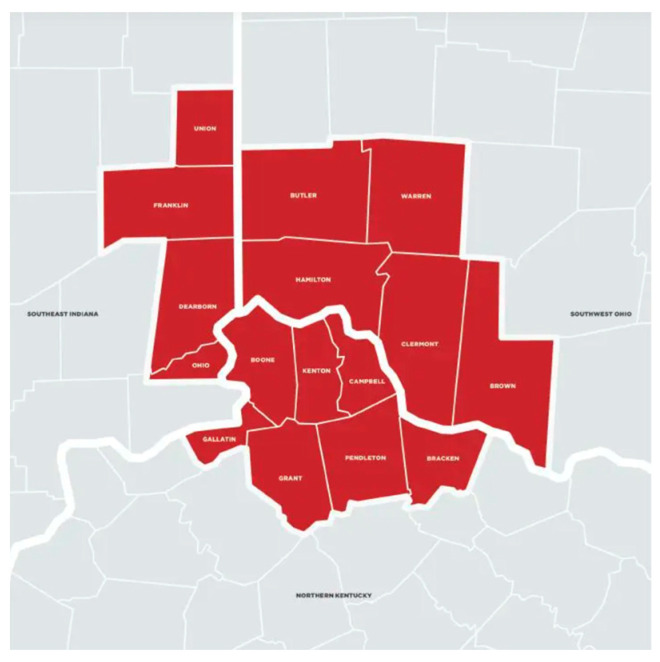
Map of the Cincinnati metropolitan area.

The Cincinnati metropolitan area comprises 16 counties in Ohio, Kentucky, and Indiana (see Figure 1). Migration is increasing dramatically in Greater Cincinnati, and the Latinx population grew 91% between 2010 and 2020 [82,83]. The U.S. Census Bureau estimated that in 2020, 95,073 Latinx individuals were living in the Cincinnati metropolitan area [103]. This estimate is likely an undercount due to how the census defines residences, migration patterns, and diversity within the Latino community, which is not well captured by the structure of the census [104]. The Cincinnati Latino immigrant population is predominantly of Mexican and Guatemalan national origin, with ~25% spread among other Central and South American countries [67,77,105].

### 2.3. Data Collection

Interviews began with Latinos who were recommended by community partners, and then participants were recruited through snowball sampling [106,107], where participants recommended other Latino immigrants who might be interested in sharing their perspectives. Participants received a $20 gift card for their time. Community members of the research team conducted semi-structured qualitative interviews [108] using a guide with questions about social and community contributors and barriers to health and wellness. Participants were also asked follow-up questions based on their responses, and if responses were limited, they were prompted to think about the question as it relates to other Latinos they know in the area. Interviews lasted 30–60 min and were conducted in person or over Zoom. We conducted interviews to the point of saturation [109,110] operationalized for this study as there were no new codes/ideas in three consecutive transcripts. Interviews were audio-recorded, transcribed verbatim, and translated to English.

### 2.4. Data Analysis

We used a hybrid (inductive/deductive) analysis approach [96,98,99] to code and analyze the interviews. Overall, we followed Braun and Clarke’s established multi-step process for analysis [93,94]: familiarization with the data, generation of initial codes, searching for themes (in our case categories), reviewing categories, and defining and naming categories. We used an Excel spreadsheet to organize and manage the coding/analysis process. Our hybrid approach had three steps. Step 1 was an inductive analysis. Specifically, three members of the analytic team reviewed the same 38 transcripts and developed an initial codebook that was generated from the data. Then, the team individually coded each line of each transcript for the first 15 interview transcripts using descriptive coding and in vivo coding of participant responses [111]. The team met weekly during the coding process to discuss any discrepancies with the codes, which were resolved through discussion and consensus. Each week, the team compiled and synthesized common codes, which ultimately resulted in broad categories. Step 2 was a deductive analysis where we applied concepts from the existing literature to the inductively derived categories. When categories did not align with the existing social and community support literature, we created new categories. In Step 3, we further distilled and synthesized categories to create the final domains. For each domain, we identified illustrative quotations.

## 3. Results

We identified seven domains representing dimensions of social connection from the perspective of Latino immigrant participants:

(1) Lens of the individual
(2) Immigrant experience
(3) Interpersonal support
(4) Community belonging
(5) Community capital
(6) Community navigation
(7) Social acceptance

Two of the domains (lens of the individual and immigrant experience) are applied to all levels of a person’s life as a filter through which participants experience aspects of social connection. The other five domains each reflect a specific aspect of social connection that supports immigrant health and well-being. For each of these five domains, participant responses fell along a continuum ranging from positive aspects supporting social connection to barriers that increase the risk of disconnection. See Table 2 for a summary of the seven domains and illustrative quotations.

### 3.1. Lens of the Individual

Participants overwhelmingly described individual-level factors contributing to health and well-being. They described social and community experiences not as universal but as unique experiences colored by the individual personality, traits, and disposition of the individual. Individual attributes influence the degree to which participants engage with other people and their broader communities; for example, introversion, willingness to trust others, self-esteem, and personal drive. Talking about the importance of individual authenticity, one participant explained, “I have a unique personality that nobody has, and whoever wants me around must accept me”. Participants described that “having a clear sense of purpose or a strong sense of self” can help “fight through hard times”. Participants often highlighted individual-level contributors to positive mental health and well-being, including psychological resilience, faith/spirituality, and the use of healthy coping and wellness strategies (e.g., exercise, listening to music, and going outside). Psychological obstacles to health and well-being included substance abuse, depression, other mental illness, and feelings of hopelessness—all seen as “barriers put on by ourselves”.

### 3.2. Immigrant Experience

Participants indicated that the immigrant lens serves as a filter through which their entire social ecology is perceived—“there are people who accept you, and there are people who do not accept you just for being Latino or for being an immigrant. And we are going to get it everywhere. In other words, wherever you go, you are still an immigrant”. Some participants described being acutely aware of being an “outsider” and feeling far from everyone they knew and loved in their home country. Participants said that their experiences as immigrants can inform expectations about social relationships and might lead to differences in the way they engage (e.g., being afraid to talk to neighbors or accessing needed health and social services because of possible discrimination). One participant described the solace and comfort of finding other Latinos in Cincinnati: “To get to know a Latino and they speak to you in Spanish, you say, ‘Oh, yes. I have been looking all over for you’. So, then you feel relief”. Some aspects of being an immigrant, such as not speaking the same language as others, learning to live in the US, learning the laws and culture of the host country, and missing family and culture of their native country, were common to all immigrants. However, participants noted that the immigrant experience in Cincinnati is unique compared to other US locations because “as a place where we’re kinda invisible, there are few spaces for Latinos to meet”. One participant described Cincinnati as “a personal ghost town where no one looks like me. Here, I don’t see the unification because there’s not many Latinos like in Florida, California, Texas”.

### 3.3. Interpersonal Support

Participants highlighted the individual-level supportive interactions they have with others as being foundational to their quality of life. Interpersonal support can range from “small talk interactions”, referring to greeting others or chatting, to long-term, loyal friendships. Taken as a whole, these interpersonal relationships can support participants emotionally, offer help in times of need, share responsibilities and expenses, and contribute to an overall sense of social and community support—“the best way to solve your problems isn’t only with courage and will, it’s also having strong support from those around you”. Interpersonal support is not limited to face-to-face interactions but can also be provided through phone calls, texting, and social media. Participants also described negative interpersonal interactions, including bullying, social comparison, and competitiveness, leading to the need to “stay away from people who seem problematic or who cause problems”.

### 3.4. Community Belonging

A feeling of connectedness to and inclusion within the surrounding community was described as supporting health and wellness—“when someone has the feeling of belonging and that they have a good place within their tribe and are in harmony, it definitely changes one’s mood and one’s health, it changes everything, it’s the concept of being complete”. This emotional connection, inclusion, and belonging to a community does not necessarily need to come from a shared identity as a Latino immigrant. Rather, participants described a variety of ways to connect, including shared experiences as parents, shared language, in the workplace, or as an immigrant. Participants prioritize making strong and durable connections with the community and describe the ways that shared values, and identities can bolster this sense of belonging. Issues such as language barriers, discrimination, or relational aggression can get in the way, but most participants felt that they have at least one meaningful connection. Participants commonly expressed that connectedness should not require extra effort but should be available through the normal interactions of everyday life (work, school, and places you are already going). However, convenience does not mean superficial connections but built-in opportunities to feel part of the community. Participants expressed the desire for belonging and had worries about feeling disconnected or being invisible. Most participants described being able to find connections and a general sense of inclusion in the larger community. However, there was a concern about being excluded based on being a Latino immigrant in a location where the Latino population is small—“where no one looks like me”. Community connectedness was so desirable that participants expressed fears about not having those crucial connections and feeling isolated. 

### 3.5. Community Capital

Participants in this study identified trusted people and places in their social environments that offered resources, connection, and support, all contributors to health and wellness and quality of life.

*There was a library close to my work. It was a divine library and the biggest one. I really enjoyed being there. I would be tired or hungry, but I would use the computer there to communicate with my family. I’d use the library to take classes and learn how to read in English. I would take classes and then I knew more people that I related to more. I started to feel that warmth of human connection.*

Almost everyone mentioned family and informal relationships with coworkers, friends, and neighbors as sources of community capital. Churches were also frequently cited as hubs in the community network not only for social relationships but also as a place to connect to resources. Participants mentioned the importance of key advocates in the community; these “in-the-know” people serve as central nodes in the network and include community organizers, promoters, and those who are well-connected to resources, actively help community members find resources, support connections, and work to make community improvements that benefit Latinos. Participants described feeling validated when the visibility of the Latino community grows—more Latino businesses, cultural events and festivals, and Latino-serving organizations. Paid professionals (therapists, interpreters, and school staff) and other representatives of community organizations (ESL education centers, unions, nonprofits, and community centers) were called out as offering support, connection, and access to resources.

### 3.6. Community Navigation

The degree to which participants can find and access the resources they need and want in the community was identified as a salient factor influencing health and wellness. As one participant stated, “The ability of community navigation is central to having a sense of community and, ultimately, quality of life”. Resources included healthcare, employment, financial information, education, and technology. Participants explained that there are numerous barriers to accessing community resources even when they are highly motivated to seek them out. Barriers include knowing where to look for resources and what resources exist for Latino immigrants, finding providers who speak one’s native language, the technological literacy required to access online resources, and the degree of hassle and inconvenience in finding and accessing resources.

*To ask for a service, you have to jump around. I call one place, which gives me another phone number for another place, and then to another place, and another place, and another place. And if you don’t speak English, just forget it! You have to call with someone who can speak English or who can help support you there. The first answer you are going to get is that you have to stay in line, and then they ask you for an email. To get the resource, you have to have an email address. So, I consider myself extremely fortunate that I can give an email address and that I can find that help, but not all Latinos have that capability.*

When it comes to community navigation of resources, participants felt out of the loop and that they did not know the “right people” to ask for guidance and were not in the right social networks conducive to navigation (e.g., word of mouth, referrals, and recommendations). Participants said that the resources they can locate are often not well-suited to their unique context as immigrants. Participants noted differences in their quality of life depending on how successful they are in community navigation. Explaining the challenges associated with community navigation, a participant said, “There are places where Latinos can support themselves, but there is a lack of information. So that is a limit. There are no ways to spread the information of getting help, and that’s what is needed”. Another participant described the difficulties of navigating resources as a new arrival: “When you arrive here, there is no information about a hospital or where a nearby supermarket is. There is no information about a care center or where you can go or where the bus is. No one puts information on a card or something, and it’s not like you can go on the street and you see a hospital sign nearby”.

*In Cincinnati, there are groups of different races and other groups that are superior. It makes me feel less for being Latina. It’s something that has happened to me… especially when I go to events alone, and I’m the only Latino; I feel that I’m not the same. But when I am in my community, I feel the same as everyone; I feel included. When I go to other events, I feel different, like I’m not supposed to be there.*

### 3.7. Social Acceptance

Feeling accepted by the Latino community, the broader community (school, neighborhood, and workplace), and society was a key contributor to positive health and wellness. Feeling accepted was not just a matter of others being friendly; it was a deeper sense of feeling welcomed and wanted. Participants explained that feeling accepted and welcomed in the community not only includes casual interactions but also the infrastructure that supports immigrant inclusion (e.g., signs in one’s native language, Latino stores, agencies, and restaurants). A few participants described disruption to feeling accepted, including racism, discrimination, and the perception that Latino immigrants take jobs and other resources. These disruptors led to a sense of social rejection, making participants feel like “outsiders”.

## 4. Discussion

**Figure 2:**
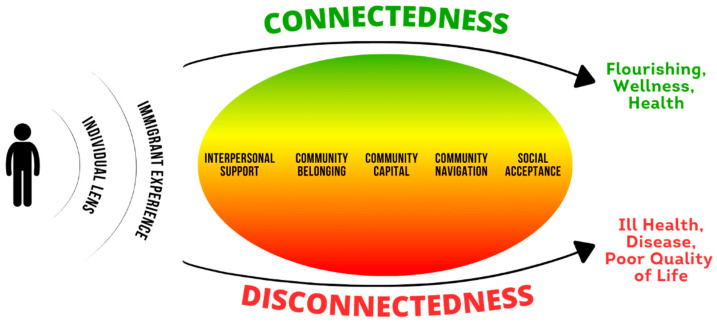
Illustrated summary of qualitative results.

Social connectedness is recognized as a core dimension of health and wellness [39,112,113], yet the way social factors intersect or act independently to influence health is not yet understood, particularly for immigrants. Social connection is more than the number of close relationships; it also includes the structure, function, and quality of interactions within and across multiple contexts [91,114]. Bronfenbrenner and other researchers have described interactions and influences on health at the individual, relationship, community, and societal levels as the social-ecological model [27,28,29,30,31,32]. In this qualitative interview study, Latino immigrants living in a nontraditional migration area described the social connection factors that are most relevant to their health and well-being. We identified seven domains that we illustrate in a model of social connection, in which health and wellness are influenced first by an individual lens and immigrant experience, then through multiple relational (interpersonal support), community (community belonging, community capital, and community navigation), and societal (social acceptance) dimensions. Figure 2 depicts the individual and their immigrant experience within the broader context of social connection. The five dimensions of connectedness are not dichotomous; rather, each dimension occurs on a continuum and contributes uniquely to health outcomes. For example, a Guatemalan immigrant who migrated with their family and speaks an indigenous language might have strong interpersonal support but low community belonging and social acceptance. To develop effective health intervention and prevention efforts for immigrants, we must be able to identify aspects of social connection that might function as protective or risk factors.

Latino immigrants in our study frequently discussed individual-level factors that influence social connectedness, including the degree to which an individual is extroverted, trusting, or motivated to be part of a group. Participants tended to perceive their own experiences and characteristics as the most prominent influence on well-being. These results are consistent with social-ecological theories of health, which center individual-level factors within nested circles of influence [92] and draw attention to the impact of individual differences in how people interact with others. Much of the immigrant health literature has focused on individual-level factors like acculturation [115] and documentation status [46,116], but Latino immigrants in our study tended to perceive these factors as important but secondary to the individual experience. In other words, immigration factors are significant social determinants of health but must be considered within the context of the individual. Although obvious, the implications for immigrant health research are clear and important: people must be understood as individuals first, immigrants second.

At the relational level, interpersonal interactions with family, friends, neighbors, and even strangers were all considered potential sources of support that could allow Latino immigrants to feel a sense of social connection and, therefore, a better quality of life. The prominent protective role of connections to family and friends among Latino immigrants is well-supported in the literature [51,117,118]. Interpersonal relationships of any kind can provide various types of support, which have all been linked to positive health outcomes [119]. More surprising was the inclusion of interpersonal interactions with acquaintances or strangers as potential sources of social connection. Even one-off experiences with another person online or in vivo were identified as supportive. Health research has provided clear evidence that “strong ties” with close friends and family provide crucial support [91], but social psychologists have recently demonstrated that even friendly interactions with “weak ties” can contribute to belongingness and promote well-being [120,121].

At the community level, participants described several dimensions that influence connectedness. Previous literature has demonstrated that connection to one’s community has been associated with neighborhood safety, trust, volunteerism, economic prosperity, community resilience, and access to services and supports [39,91]. Being strongly community-connected and integrated within one’s own ethnic enclave may serve as a protective factor for immigrants against outsider discrimination and thereby facilitate positive physical and mental health, well-being, engagement in health-promoting behaviors, and decreased mortality [122,123,124,125,126]. Social disconnection has been associated with isolation and loneliness due to limited or inadequate meaningful connections with others [113]. Immigrants, in particular, may be at risk for social isolation and marginalization because of changes in their social support systems and not feeling like they belong in their host country due to language, perceived discrimination, etc. [49,127]. Furthermore, there appear to be significant health consequences in a dose–response relationship, such that social disconnection and lack of belongingness are associated with decrements and disparities in physical and mental health for vulnerable and minoritized groups such as immigrants [37,38,39,40]. The existing literature provides ample evidence for the importance of social connection on health, but the specific components that are most salient for health and well-being among immigrants are not well understood.

Latino immigrants in this study described three specific aspects of connectedness at the community level that they perceived as being most salient for health and well-being. First, a sense of belonging, whether due to shared Latino immigrant identity or aspects of work, parenthood, or daily life, contributed to participants feeling welcomed and included in their communities. Participants in this study were adamant that community belonging should be convenient and not require additional effort—in other words, there should be built-in opportunities for community connection in everyday life. A few participants were worried about invisibility and being disconnected in their communities because of the small number of other Latinos. Belongingness is a basic psychological need and has been significantly associated with feelings of safety and security, life satisfaction, well-being, and positive mental health [128]. Lack of belonging can have detrimental consequences impacting a range of both physical and mental health issues, including [129,130,131,132,133]. Interestingly, one recent study of Latino mental health during the COVID-19 pandemic found that mental health disparities are less attributable to Hispanic ethnicity than to belongingness to the dominant culture [131].

Trusted people and places in the community, or what we refer to as community capital, were viewed by participants as essential to security, obtaining what you need, and increasing quality of life, all of which ultimately were perceived as strengthening connectedness. Again, the idea of visibility came up but as related to growth in Latino community infrastructure (e.g., Latino businesses, cultural events, and Latino-serving organizations). Predicting better mental and physical health, serving as a protective factor against mortality, and associated with community resilience, social capital can be understood as social cohesion, social networks, social trust, reciprocity, and civic engagement [134,135,136,137]. Attempting to avoid the conceptual imprecision of the term social capital, we use ‘community capital’ to emphasize trusted resources within the place and context where immigrants live, work, and pray [67]. Lack of community capital can negatively impact health. For instance, people living in disadvantaged neighborhoods may not experience health advantages associated with strong social networks and have limited opportunities for social connection and access to resources due to underfunded community settings, crime, and poverty [138,139].

Participants in this study identified navigation to locate and access resources as an important, albeit atypical, component of community connectedness. The hassle factor and inability to find Latino-tailored resources were noted as major barriers that interfered with participants’ sense of community, stability, and life satisfaction. Community navigation can be viewed as a source of informational support, which has been linked to lower mortality and better health [140]. Our results suggest that community health workers or *promotores*, who are commonly employed to connect Latinx immigrants to resources [141], have the potential to indirectly promote health by increasing community connectedness. *Promotores* in Pittsburgh, another nontraditional migration city, were shown to increase not only access to resources but also perceived social support [142].

Latino immigrants described infrastructure, policies, and other aspects of their environment that made them feel either more or less accepted by society. In terms of ecological theory, these factors that do not directly involve the individual yet still impact well-being are at the utmost layer of one’s ecological system. Participants described infrastructure that promoted social acceptance, like signs and access to social and healthcare services in their native language, as well as societal factors that are exclusionary, like racism and discrimination in state and federal policies and anti-immigration sentiment in our country. Participant responses are consistent with research evidence documenting the negative impact of law enforcement policies and federal immigration policies on the mental health of Latino immigrants [143,144]. Notably, societal-level policies and infrastructure are not the only barriers to social acceptance. Participants also described positive interpersonal interactions and community-level influences (e.g., faith-based communities and Latino festivals) that could bolster perceptions of social acceptance.

Several limitations of the current study suggest avenues for future research. To our knowledge, our study is the first to conceptualize social connectedness as experienced within multiple ecological systems by Latino immigrants. We used non-probability sampling to recruit 38 participants from a nontraditional migration area, limiting generalizability. Thus, findings may not apply to other Latino immigrant populations living in ethnic enclaves in cities with an established history of migration. Future research should expand the investigation of social connections between immigrants living in diverse geographic areas and other immigrant groups to fine-tune the aspects of connectedness driving immigrant health.

Our qualitative research uniquely contributes to the literature by identifying the aspects of social connection that Latino immigrants in a nontraditional migration area believe influence health and wellness. Unlike quantitative surveys that start with a prioritized concept and ask participants to rate their experiences, our project began with Latino immigrants asking other Latino immigrants to describe the social factors that matter in their lives. Socioecological models conceptualize the role interpersonal, community, and societal systems (and the interplay between systems) have on health outcomes [27,28,29,30,31,32]. Our results provide the specific social factors within each of those systems that Latino immigrants identify as driving their own health outcomes. Additionally, our research contributes to the literature by elucidating the social connection factors most relevant to immigrants living in nontraditional destination areas. Most of what researchers know about Latino communities is based on participants in traditional migration areas (e.g., Florida, California, and Texas), but the social-ecological context in these ethnic enclaves is vastly different from the experiences of Latinx immigrants in the Midwest and South, where Latinx immigrants increasingly live [145]. Our results can inform future research and intervention efforts seeking to improve health outcomes in areas where immigrants are at high risk of isolation and social disconnection.

## 5. Conclusions

Previous literature has highlighted that social and community influences can contribute to positive health outcomes among immigrants, but these factors have been studied in isolation, and related concepts are vague, overlapping, and conflated. We lack conceptual clarity of the distinct connectedness domains that drive positive health outcomes in immigrant populations. Additionally, most immigrant health research relies on samples in established migration areas, where the risk for social disconnection is lowest. Social connection has been identified by the U.S. Surgeon General as a public health crisis [82], yet we currently lack a comprehensive way to assess these concepts in diverse immigrant communities. Our qualitative, community-generated understanding of social connection might serve as a foundation for a quantitative measure that can be used by providers to assess the connectedness of their patients and by researchers to evaluate the effectiveness of community-level interventions with immigrants.

## Figures and Tables

**Table 1:** Participant demographic information (N = 38).

Demographic Characteristic	Frequency	Percent
Country of Origin		
Guatemala	11	29%
Mexico	9	24%
Venezuela	9	24%
Colombia	2	5%
Honduras	2	5%
El Salvador	2	5%
Dominican Republic	1	3%
Ecuador	1	3%
Panama	1	3%
Current Location		
Ohio	29	76
Kentucky	8	21
Indiana	1	3
Children		
Yes	24	63
No	8	21
Not Reported	6	16
Age		
18–30	11	29
31–40	13	34
41–50	7	18
51–60	3	8
61–71	3	8
Not Reported	1	3
Years Living in the USA		
Less than 1 year	4	11
1 year	6	16
2–10 years	11	29
11–20 years	7	18
21–30 years	5	13
31–40 years	2	5
Gender		
Female	22	58
Male	16	42

**Table 2:** Summary of domains and illustrative quotations.

Domain	Example Quotations (Translated to English)
Lens of the individual	“Well, yes, of course someone can get out of hard situations. Someone has to fight for it and to stay positive to be able to get up after a negative situation.” “The thing is that I try not to bother other people, and I cover my own necessities…so I don’t have to sit there and ask over and over again for help. That’s not me.” “If you put your mind to it…we can achieve anything. I think the barriers we have are put on by ourselves.”
Immigrant experience	“You identify yourself in the place where you are, but there is a lot I do not know…one is always waiting to answer the question of ’is this my place?’” “It’s hard for Hispanics to communicate in a purely American environment. If I knew English, I would communicate more with my American neighbors. I can’t really have a strong relationship with Americans because of the language barrier.” “As Hispanics, we are a little afraid as many of us don’t have documents or can’t work here legally. Not all Hispanics feel supported.”
Interpersonal support	“Happiness, we try to establish it, not just by focusing on work and getting home but by taking time as a family, having dinner together, talking about how our day went. We try as a family to be more united.” “Social media, I use it in a good way to talk to my family, although it’s not the same as talking face to face, but they have helped me a lot, and my friends have helped, too…I have two group chats, and we talk there and give each other advice.” “I am retired. So, when you’re retired, your circles shrink…volunteering permits me to meet other people of all kinds with whom I did not have connections before. We would have never crossed each other’s paths, so it’s a very enriching experience.”
Community belonging	“In places like where we play soccer, everyone gets together… doing some kind of celebration like a birthday celebration. The networks are there, and they are connected, and they do trust each other.” “Some don’t like immigrants in their country, but here in Cincinnati, we have had a very good welcome, and there are many people who have welcomed us with open arms who are willing to help us, and that makes us happy. It makes us feel good. I can’t imagine feeling rejection because it brings negative emotions or the emotions that make us feel bad and make us sick.” “I feel connected more to the community in Cincinnati. The community in Indiana is a very separate community. Everyone is in their own world; people are just with their work, people are with their families. But I don’t feel connected to any one of them.”
Community capital	“I think social support is when someone is connected with a place or institution that one trusts…there isn’t one place that can help with everything, but this place of trust could refer one to other resources.” “I use Facebook because here in the United States, they use this app a lot to find information, like a marketplace to buy things, and I also use it because I can see cultural events and family events that are happening nearby, or if there is a park nearby to take my son.” “Twenty-three years ago, there was nothing…it was very, very difficult, but nowadays there is a lot of help, and I know that there are people who benefit…Even if you don’t speak the language, there’s always someone to advise or guide you.”
Community navigation	“This community is very welcoming. Not everyone will open doors to others, not physically, but they at least guide them to where they need to be.” “I think it’s all the same unknowns and search for a center—an area where someone can say, ‘this is an area for Latinos’ so they can live without fear, for people who don’t know how to communicate, to help find information such as how to get help either to get a job or find housing.” “ I could not get any communication with anyone about where they would recommend for us to go. It is very difficult to be in this country, to provide for our kids, to take our kids to the doctor. It’s very hard to get medical help when we are sick.”
Social acceptance	“Normally, when I deal with Latinos, they tell me the same thing. That they are alone and that they don’t feel included or accepted. More than anything, they don’t feel accepted. I think it depends a lot on the economy; the less your finances are, the less accepted you are. The better your finances are, the more you will be accepted. And it also has to do with physical features and what country you are from because every country has a different treatment in Latin America. Some worse than others.” “I have friends who suddenly feel that others look at them differently because they’re Latina, but it has never happened to me…here if you go to the supermarket, people are respectful and say good morning and thank you. I think that is a part of being accepted and seen in a good way and that you feel that you are part of a society.” “I think that one can feel accepted here when one learns how to live here, respects the laws here, and learns the differences of how others live. We need to learn that Americans have another way of living and accept and respect that. One needs to try to communicate in English and acculturate.”

## Data Availability

Data are contained within the article.
